# Annotations

**Published:** 1904-04-23

**Authors:** 


### ANNOTATIONS.

### Sir Henry Thompson.

By the death of Sir Henry Thompson not only the medical world, but the social world in general, has suffered a loss which is practically irreparable. Sir Henry Thompson was an example of successful versatility, which must become rarer as the cult of specialism advances. He commenced his brilliant medical career in 1848, soon became a Gold Medallist, obtained many prizes, and passed through various stages of the medical career at University College Hospital. He became Emeritus Professor of Clinical Surgery in 1875, and was created a baronet in 1899. He has a place in history through his attendance on the late King of the Belgians and the last Napoleon, who both suffered from stone. Thompson had studied diligently under Civiale in Paris, and was especially conversant with the latest methods of treating this disease. But distinguished as was his medical career, Sir Henry Thompson's social qualities were equally brilliant. He was a talented artist and exhibitor at the Royal Academy, a writer of fiction, an art collector, and a talented host. His advocacy of cremation has done much to secure its adoption. Apart from his professional income, he became a man of considerable fortune through a legacy from a grateful patient.

### The Care of the Insane Poor.

The "Care of the Pauper Insane in Scotland and its Cost" is the title of a paper in the current number of the *Westminster Review* by Mr. James Motion, and in the article strictures of the present system are somewhat severely dealt out to the responsible authorities. It is well known that in most points the Scottish lunacy system is in advance of that of England, and therefore Mr. Motion's strictures apply with additional force to the English system. The author shows that in the Glasgow district alone the cost of caring for the insane had gone up from £19,000 in 1871, to £68,000 in 1901. Further, we are told that the Royal Asylums of Scotland have ceased to discharge their original functions to the poor insane, and that they now cater "for the wealthier classes of the insane from all quarters of the country," and as a consequence the patients for whom they were intended are being driven to pauper asylums. This is a severe indictment, and, if it can be substantiated, it detracts immensely from the usefulness of these institutions; and, indeed, makes them run on parallel lines with many of our English lunatic hospitals, whereas we had cherished the belief that these Royal asylums were really doing the work that they were intended for. The cost of the district asylums now ranges from £300 to £600 a bed, and the "general board I hold to be responsible for the extreme and lavish provision for the insane." As to the accumulation of the chronic insane in asylums, Mr. Motion says it is "largely owing to the difficulty of getting the hearty co-operation of the superintendents in sending out such cases." We doubt if the last statement can be quite correct, as medical superintendents, as a rule, would be only too thankful to purge the wards of these chronics; but in any case the ratio of pauper lunatics boarded out in Scotland is enormously greater than it is in England. We have in these columns more than once called attention to the evils of large asylums: to the increased and ever increasing cost of the building of pauper asylums, and to our lunatic hospitals "catering" for high-paying patients, and we should welcome almost any innovation whereby these evils, or any of them, could be lessened. The transference of the Lunacy Administration to the Local Government Board has been suggested, and it is conceivable that some good might result from the change. Whatever change be effected in England, it is much to be desired that the Visiting Commissioners in Lunacy be all medical men and all known as successful medical superintendents of county asylums. What would the legal profession say if three fourths of the county-court judgeships were given to medical men?

### Myopia and School Life.

At the recent International Congress of School Hygiene held at Nuremburg an important address dealing with the relationship of the development of myopia to the conditions of school life was delivered by Professor Cohn of Breslau. Based upon an experience extending over some forty years Professor Cohn has found that the number of short-sighted children increases with the development of the school, from the lowest village school to the gymnasium; and that the proportion of myopic children, and also the degree of the myopia, steadily increases from the lower to the higher classes. He thus regards myopia as a widespread disease produced by school life. Whilst the essential cause of short sight cannot be confidently stated, it is certain that much close work, especially when associated with hereditary disposition and bad lighting, is the circumstance which acts as an exciting cause. The prevention of myopia therefore comes within the scope of school hygiene. The position of the writing table and the manner in which the pen is held have been shown to be of great importance, and when these points are attended to myopia is found to be less frequent. The use of properly-printed books is another point which needs attention. Of great influence also is the lighting of the school-room, and this is often found to be seriously defective. All these considerations become of great importance when it is remembered that a myopic eye is a diseased eye and that the number of those who suffer from this condition is an increasing one. Professor Cohn's experience can find many parallels in this country, and his proposal of "no school without an ophthalmologist" will meet with many sympathisers. Unfortunately there is a strong popular prejudice that short sight is strong sight, and many educational authorities are far from being alive to their responsibilities in this department. The question ought to be pressed upon the attention of teachers and others having the care of children. This can be done not only by ophthalmic surgeons and the medical officers of large schools. Every private practitioner should see that myopic parents are made alive to the special risks of their children, and thus take his part in establishing a sound public opinion where there is at present much ignorance and prejudice.

